# Effects of Dance Interventions on Cognition, Psycho-Behavioral Symptoms, Motor Functions, and Quality of Life in Older Adult Patients With Mild Cognitive Impairment: A Meta-Analysis and Systematic Review

**DOI:** 10.3389/fnagi.2021.706609

**Published:** 2021-09-20

**Authors:** Chang Liu, Mengyu Su, Yuchen Jiao, Yan Ji, Shuqin Zhu

**Affiliations:** School of Nursing, Nanjing Medical University, Nanjing, China

**Keywords:** mild cognitive impairment, dance, elderly, system review, meta-analysis

## Abstract

**Background:** Dance interventions are considered beneficial for older patients with mild cognitive impairment in many aspects. We conducted a comprehensive systematic review and meta-analysis to assess the effects of dance on different aspects (cognitive function, emotions, physical function, and quality of life) of this population.

**Methods:** A systematic search of PubMed, Web of Science, the Cochrane Central Register of Controlled Trials, Embase, American Psychological Association PsycInfo, ProQuest, Scopus, Cumulative Index to Nursing and Allied Health Literature, the Chinese BioMedical Literature Database, the VIP Database for Chinese Technical Periodicals, China National Knowledge Infrastructure, and Wanfang Data database was performed. Two reviewers independently assessed the study quality.

**Results:** Fourteen studies were retrieved from the databases for analysis. The pooled results showed that dance interventions significantly improved global cognition (standardized mean difference [SMD] = 0.73, 95% confidence interval [CI]: 0.47 to 0.99, *P* < 0.00001), rote memory (mean difference [MD] = −2.12, 95% CI: −4.02 to −0.21, *P* = 0.03), immediate recall (SMD = 0.54, 95% CI: 0.30 to 0.78, *P* < 0.0001), delayed recall (SMD = 0.56, 95% CI: 0.26 to 0.86, *P* = 0.0002) and attention (SMD = 0.38, 95% CI: 0.13 to 0.64, *P* = 0.003). No significant improvement was found in executive function, language, depression, anxiety, dementia-related behavioral symptoms, motor function, and quality of life.

**Conclusion:** Dance interventions benefit most aspects of cognitive functions. The evidence for the effects of dance on psycho-behavioral symptoms, motor function and quality of life remains unclear. More trials with rigorous study designs are necessary to provide this evidence.

## Introduction

The current concept of mild cognitive impairment (MCI) was first proposed by Reisberg et al. (1988), to describe a transitional phase from the cognitive changes occurring in normal aging to those typically found in dementia. *The Lancet* reported that approximately one-fifth of the older population, aged over 65 years, experience MCI (Livingston et al., 2017). Patients with MCI have a higher risk of dementia than those without MCI. The rate of dementia in MCI patients is 10–15% per year, whereas in healthy controls it is only 1–2% per year (Li et al., 2016). For patients with MCI, there is a high risk of degeneration of physical functions (Ehsani et al., 2020) and an upsurge in negative emotions, such as depression (Ma, 2020). They also experience irreversible physical and psychological complications and the gradual loss of social attributes and personality. The family caregivers of patients with MCI face financial hardship, a considerable burden of care, and an emotionally traumatic experience with social isolation—watching their relatives become “familiar strangers.” MCI is the golden period of dementia prevention. Early intervention should be performed at this stage to slow down the development of dementia and to bring about cognitive reversal (Zou et al., 2019).

The intervention methods for MCI can be pharmaceutical or non-pharmaceutical. Current reviews have found that acetylcholinesterase inhibitors (such as donepezil and galantamine) used to treat Alzheimer's disease (AD) have proven ineffective in the MCI treatment group (Bachurin et al., 2018). However, drug interventions cause significant side effects. In contrast, non-pharmaceutical interventions, including cognitive intervention, psychotherapy, and physical activity have attracted significant attention owing to the lack of drug-induced side effects. Physical activity has particularly shown good applicability in patients with MCI (Langa and Levine, 2014), and is a preventive and possible protective factor for dementia (Livingston et al., 2017). It can improve cognition by improving blood perfusion, increasing endorphin and serotonin levels, and improving neuroplasticity (Kropacova et al., 2019). The activities with the most potential benefits are multi-component exercises (Cai and Abrahamson, 2016). Multi-component exercises can improve overall cognition and executive function and positively affect memory and brain activation (Lam et al., 2018), especially dance movements involving the body, combined with recognition knowledge and social stimulation (Burzynska et al., 2017).

Due to its inherent expressiveness, creativity, and aesthetics, dance is often used as a popular MCI physical activity intervention method. Furthermore, a literature review has shown that dance intervention had a positive effect on the population's cognition, physical functions (such as sensorimotor ability, gait posture, balance, and endurance), lifestyle, emotions, and social functions, thereby improving the quality of life (QOL) of the research subjects (Kattenstroth et al., 2013). A study involving “social” exercise protocols—a period of 6 months (60 min/1 time/week) of dance intervention—has found significant improvement in global cognition, attention, memory, visual-spatial ability, and language ability of the elderly. Studies have shown that dance intervention, as a cognitive stimulus, can stimulate different neural structures to improve various cognitive domains. Subjects can simultaneously learn and memorize new movement patterns, pay attention to follow instructions, execute complex movement patterns, and express emotions and social interactions to stimulate the neural structures that rely on various cognitive functions and improve their neuroplasticity (Hewston et al., 2021). However, another dance intervention study for patients with MCI has found that after 3 months of moderate-intensity aerobic dance, the patients' executive function did not improve significantly (Zhu et al., 2018).

A meta-analysis has shown that compared with other forms of exercise intervention, dance intervention has a specific positive effect on improving physical functions, such as muscle strength, balance, and flexibility (Fong et al., 2020). It also has a specific effect on improving participants' cardiovascular function. Dance can be used as a safe and effective alternative to exercise, reducing the physical health risks and outcomes associated with sedentary and inactive behaviors. Moreover, existing evidence proves that dance intervention can reduce the risk of falls to a certain extent (Liu et al., 2021; Witkoś and Hartman-Petrycka, 2021). Thus, it has a particularly positive effect on improving physical functions, such as muscle strength, balance, flexibility, and it also has a particular effect on improving the cardiovascular function of participants. Nevertheless, Qi et al. have found that after 3 months of moderate-intensity aerobic dance intervention, the balance score of the intervention group was not significantly higher than that of the control group (Qi et al., 2019).

The progression of the dance intervention requires the two brain hemispheres to operate simultaneously while stimulating motor awareness, logic, music, and emotional processes (Douka et al., 2019). The European Association for Dance Movement Therapy believes that creative expression and communication are core components of dance sports therapy. Creative art therapy can positively affect mental health through the mechanisms of body and mind, communication, and emotional expression (Liu et al., 2021). Additionally, dance is usually performed in a social, pleasant environment, improving patient mood, reducing loneliness, and showing psychological benefits (Carapellotti et al., 2020). Group dance is a form of social activity rooted in human culture (Witkoś and Hartman-Petrycka, 2021). People can achieve social satisfaction by better recognizing themselves in groups with similar interests and goals, reducing social isolation, improving patient social function, and realizing social satisfaction.

The influence of dance intervention on patients with MCI is mainly manifested in the following aspects: cognition, psycho-behavioral symptoms, motor functions, and QOL. Systematic reviews have been published on the effect of dance intervention on patients with MCI; current systematic reviews have reached a consensus on the positive effect of dance intervention on global cognition (Chan et al., 2020; Meng et al., 2020; Zhu et al., 2020; Hewston et al., 2021; Muiños and Ballesteros, 2021). Chan et al. have found (Chan et al., 2020) that dance can improve the memory function of the older patients with MCI; the study by Zhu et al. confirmed this (Zhu et al., 2020). Two reviews, including but not limited to dance interventions for MCI populations, have contrasting opinions on executive function. Meng et al. reported that dance intervention has no meaning for the executive function of the elderly (including patients with MCI) (Meng et al., 2020), whereas another study on middle-aged and older adults has suggested that dance can improve executive function (Muiños and Ballesteros, 2021), consistent with that of Zhu et al.'s study (Zhu et al., 2020). Only one systematic review has found that dance intervention can improve the attention of patients with MCI (Chan et al., 2020). Existing reviews mainly focus on the effect of dance intervention on the cognitive functions of patients with MCI, and there are significant differences. Outcomes, such as physical function, mood, and QOL, are rarely included in the analysis. It is noteworthy that Chinese scholars have made specific achievements in this field in recent years, but their work has not been included in the published systematic review analyses.

This systematic review aimed to summarize and critically evaluate dance intervention studies in patients with MCI aged over 60 years; to explore the effects of dance on the elderly with MCI based on four aspects (cognitive function, emotion, physical function, and QOL); to establish a general summary of the existing literature; and to provide a basis for follow-up studies in this field.

## Methods

This work adhered to the Preferred Reporting Items for Systematic reviews and Meta-Analyses guidelines and was prospectively registered with the International Prospective Register of Systematic Reviews (CRD42021230159).

### Search Strategy

We searched the following electronic bibliographic databases: PubMed (PubMed, RRID:SCR_004846), Web of Science, the Cochrane Central Register of Controlled Trials (Cochrane Central Register of Controlled Trials, RRID:SCR_006576), Embase (EMBASE, RRID:SCR_001650), American Psychological Association PsycInfo (PsycINFO, RRID:SCR_014799), ProQuest (ProQuest, RRID:SCR_006093), Scopus, Cumulative Index to Nursing and Allied Health Literature, the Chinese BioMedical Literature Database, the VIP Database for Chinese Technical Periodicals, China National Knowledge Infrastructure, and Wanfang Data. To ensure the comprehensiveness of the included studies, we used combinations of Medical Subject Headings (MeSH, RRID:SCR_004750) and free text words without language restrictions from inception to November 12, 2020. We also retrieved the reference lists of all eligible studies and other relevant studies using alternative approaches (e.g., Google Scholar [Google Scholar, RRID:SCR_008878]). The complete search strategy is provided in Supplementary Material.

### Selection Criteria

Studies were included in the review only if they met the following criteria:

#### Participants

Diagnosed with MCI by any definite, precise, and concrete diagnostic criteria; aged 60 years or older; and able to dance or exercise independently.

#### Interventions

Dance as an intervention that is not limited to any particular type (e.g., aerobic dance, Latin, ballroom dance, Chinese square dance, and Yangko); dance not limited to the frequency or duration of the intervention; dance performed under the guidance and supervision of trained professionals or by the participants themselves.

We defined dance intervention by the following principles: systemic movements of the body that are needed to be observed and imitated, are focused on physical flexibility and expression of emotion, and are accompanied with music.

#### Comparisons

Including but not limited to drug treatment, regular therapy, and educational programs.

#### Outcomes

At least one measure of cognitive function (e.g., memory function and attention), motor function (e.g., balance and functional mobility), psycho-behavioral symptoms (e.g., depression and anxiety), and QOL.

##### Cognition Evaluation

Studies that included the following tests were included in the review: Mini-Mental State Examination (MMSE) and Montreal Cognitive Assessment (MoCA) to assess the global cognition level; Trail Making Test Parts A (TMT-A) mainly to evaluate rote memory of patients; Trail Making Test Parts B (TMT-B) primarily to assess executive function; Logical Memory I (LM-1) and Rivermead Behavioral Memory Test (RBMT) to measure immediate recall; Logical Memory II (LM-2) to assess delayed memory; the Symbol Digit Modalities Test (SDMT) or Test of Everyday Attention (TEA) to assess attention; the Boston Naming Test (BNT) to assess the language function in patients with cognitive impairment.

##### Psycho-Behavioral Symptom Evaluation

Studies that included the following tests were included in the review: Psycho-behavioral symptoms evaluated using Neuropsychiatric Inventory (NPI) to assess the 12 behavioral disorders occurring in patients with dementia such as delusions, hallucinations, agitation, dysphoria, anxiety, apathy, irritability, euphoria, disinhibition, aberrant motor behavior, night-time behavior disturbances, and appetite and eating abnormalities; degree of anxiety mainly measured by the Hospital Anxiety and Depression Scale (HADS); the Geriatric Depression Scale (GDS-15, GDS-30) and HADS to test depression.

##### Motor Function Evaluation

Studies that included the following tests were included in the review: Berg Balance Scale (BBS) to evaluate balance; the Timed Up and Go (TUG) test to test functional mobility.

##### Quality of Life Evaluation

Studies that used the following two scales for QOL evaluation were included in the review: Quality of Life in Alzheimer's Disease (QOL-AD) and the 36-item Short Form Health Survey (SF-36).

#### Design

Studies that were randomized controlled trials (RCTs) or quasi-experimental trials were included in this review.

### Study Selection and Data Extraction

Two reviewers (LC, SMY) worked independently to identify studies that met the inclusion criteria briefly by screening the abstracts. If the abstract did not provide sufficient information, the full text was obtained to determine the study's eligibility for inclusion in this review. Any disagreements were resolved after discussions with a third reviewer (JYC). We excluded studies for which complete information could not be obtained despite our best attempts.

For each eligible study, information about the first author's name, country, number of participants, age, male to female ratio, control group intervention, intervention characteristics (including frequency, intensity, duration, and type of intervention), and outcome measures were extracted using a self-designed standardized form (Table 1). Two review authors (LC, JYC) extracted data independently using reference management software such as EndNote (EndNote, RRID:SCR_014001) and NoteExpress.

**Table 1:** Characteristics of included studies.

**Study**	**Country**	**Year**	**Total number (EXP/CON)**	**Age (year) (EXP/CON)**	**Proportion of females (EXP/CON)**	**Control**	**Intervention**				**Outcome measure**
							**Frequency**	**Intensity**	**Duration**	**Type**	
Adam et al. (2016)	Malaysia	2016	44/40	>60	52.3%/47.5%	Relaxation Exercises	2 days/week for 6 weeks	NA	60 min/day	poco-poco dance and relaxation exercises	① Global Cognition/MMSE ② Anxiety/HADS ③ Depression/HADS ④ Quality of Life/QOL-AD
Aguiñaga (2016)	USA	2017	10/11	76.0 ± 6.0/74.9 ± 6.8	80.0%/72.7%	Wait-List	2 days/week for 16 weeks	Light	25 min/day	BAILAMOS© program (Latin Dance)	① Immediate Recall/LM-1 ② Delayed Recall/LM-2 ③ Rote Memory/TMT-A ④ Executive Function/TMT-B ⑤ Attention/SDMT ⑥ Depression/GDS-15 ⑦ Quality of Life/QOL-AD ⑧ Functional mobility/TUG
Barnes et al. (2013)	USA	2013	31/32	71.7 ± 5.5/73.9 ± 6.3	67.7%/62.5%	NA	3 days/week for 12 weeks	Moderate	60 min/day	standard dance-based aerobics format	① Rote Memory/TMT-A ② Executive Function/TMT-B
Bisbe et al. (2020)	Spain	2019	17/14	72.88 ± 5.60/77.29 ± 5.16	52.9%/50%	Physiotherapy	2 days/week for 12 weeks	Light-to-Moderate	60 min/day	Choreography	① Global Cognition/MMSE ② Immediate Recall/LM-I ③ Delayed Memory/LM-II ④ Rote Memory/TMT-A ⑤ Executive Function/TMT-B ⑥ Language/BNT ⑦ Anxiety/HADS ⑧ Depression/HADS ⑨ Quality of Life/SF-36 ⑩ Balance/BBS Functional mobility/TUG
Doi et al. (2017)	Japan	2017	55/63	75.7 ± 4.1/76.0 ± 4.9	50.7%/46.3%	Health Education	1 day/week for 40 weeks	NA	60 min/day	Ballroom Dance	① Global Cognition/MMSE ② Rote Memory/TMT-A ③ Executive Function/TMT-B
Dominguez et al. (2018)	Philippines	2018	101/106	68.8 ± 5.6/69.4 ± 6.1	84.2%/74.5%	NA	2 days/week for 48 weeks	NA	60 min/day	Ballroom Dance	① Global Cognition/MoCA-P ② Depression/GDS ③ Language/BNT ④ Dementia-related behavioral symptoms/NPI
Lazarou et al. (2017)	Greece	2017	66/63	65.89 ± 10.76/67.92 ± 9.47	80.30%/76.19%	Wait-List	2 days/week for 40 weeks	NA	60 min/day	Ballroom Dance	① Global Cognition/MMSE, MoCA ② Depression/GDS-15 ③ Attention/TEA ④ Immediate Recall/RBMT1 ⑤ Delayed Recall/RBMT2 ⑥ Dementia-related behavioral symptoms/NPI
Qi et al. (2019)	China	2019	16/16	70.6 ± 6.2/69.1 ± 8.1	68.8%/75.0%	Health Education	3 days/week for 12 weeks	Moderate	25 min/day	Aerobic Dance	① Global Cognition/MMSE, MoCA ② Immediate Recall/LM-1 ③ Rote Memory/TMT-A ④ Executive Function/TMT-B ④ Attention/SDMT ⑤ Balance/BBS
Wang et al. (2020)	China	2020	33/33	81.06 ± 5.17/81.09 ± 7.44	78.80%/63.60%	Health Education	3 days/week for 12 weeks	Moderate	40 min/day	Chinese Square Dancing	① Global Cognition/MMSE, MoCA ② Depression/GDS-15 ③ Balance/BBS
Zhu et al. (2018)	China	2018	29/31	70.3 ± 6.7/69.0 ± 7.3	51.7%/67.7%	Health Education	3 days/week for 12 weeks	Moderate	35 min/day	Aerobic Dance	① Global Cognition/MMSE, MoCA ② Immediate Recall/LM-I ③ Rote Memory/TMT-A ④ Executive Function/TMT-B ⑤ Attention/SDMT ⑥ Depression/GDS-15
Yi (2015)	China	2016	16/16	65.8 ± 5.4/66.4 ± 5.3	68.75%/62.5%	Donepezil Hydrochloride	7 days/week for 18 weeks	Light	45 min/day	Dance Rug	① Global Cognition/MMSE
Junmei (2016)	China	2016	14/14	65.47 ± 5.21/64.51 ± 5.38	100%/100%	Donepezil Hydrochloride	7 days/week for 24 weeks	Light	40 min/day	Yangko	① Global Cognition/MMSE
Xinjian (2017)	China	2017	23/23	65.47 ± 5.21/64.51 ± 5.38	100%/100%	Donepezil Hydrochloride	7 days/week for 9 weeks	Light to Moderate	40~60 min/day	Chinese Square Dancing	① Global Cognition/MMSE
Yu (2019)	China	2019	31/32	73.4 ± 5.1/71.3 ± 6.7	83.4%/81.3%	Health Education	3 days/week for 12 weeks	Moderate	60 min/day	Chinese Square Dancing	① Global Cognition/MoCA ② Depression/GDS-30

*EXP, Experimental group; CON, Control group; MMSE, Mini-Mental State Examination; MoCA, Montreal Cognitive Assessment; TMT, Trail-Making Test; RBMT, Rivermead Behavioral Memory Test; LM, Logical Memory; SDMT, Symbol Digit Modalities Test; TEA, Test of Everyday Attention; NPI, Neuropsychiatric Inventory; HADS, Hospital Anxiety and Depression Scale; GDS, Geriatric Depression Scale; BBS, Berg Balance Scale; TUG, Timed up and go; SF-36, 36-Item Short Form Survey; QO, Quality of Life in Alzheimer's Disease*.

### Assessment of Risk of Bias of the Included Studies

Two reviewers (LC, SMY) independently assessed the study quality according to the Cochrane Handbook for Systematic Reviews of Interventions (Higgins et al., 2011). This assessment scale addresses six domains: sequence generation, allocation concealment, blinding, incomplete outcome data, selective reporting, and other issues. For each domain, reviewers performed the assessments independently and resolved differences by discussion or by appealing to a third reviewer (JYC). Each item was evaluated as “low,” “unclear,” and “high.” If the study fully met the above standards, the article quality was evaluated as A; if it partially satisfied the standards, as B; and if it was completely inconsistent with the standards, as C. The results of this assessment were summarized in both a “risk of bias” graph and a “risk of bias” summary.

### Data Analysis

We employed Review Manager 5.3 software (The Nordic Cochrane Centre, The Cochrane Collaboration, Copenhagen, Denmark) to perform the meta-analysis. Since all data were continuous, we selected the mean difference (MD) with 95% confidence intervals (CIs). When different scales were applied to measure the same outcome, we used the standardized mean difference (SMD). Heterogeneity in the included studies was tested by the Cochrane Q statistic and quantified by the I^2^ statistic. When *I*^2^ was > 50% and the *P*-values were <0.10, the study was considered to show high heterogeneity. We used the sensitivity analysis to analyze the source of the heterogeneity by excluding studies of poor quality or those whose control interventions may have exerted potential treatment effects. A subgroup analysis was also performed to explore any differential effects.

## Results

### Study Selection

**Figure 1:**
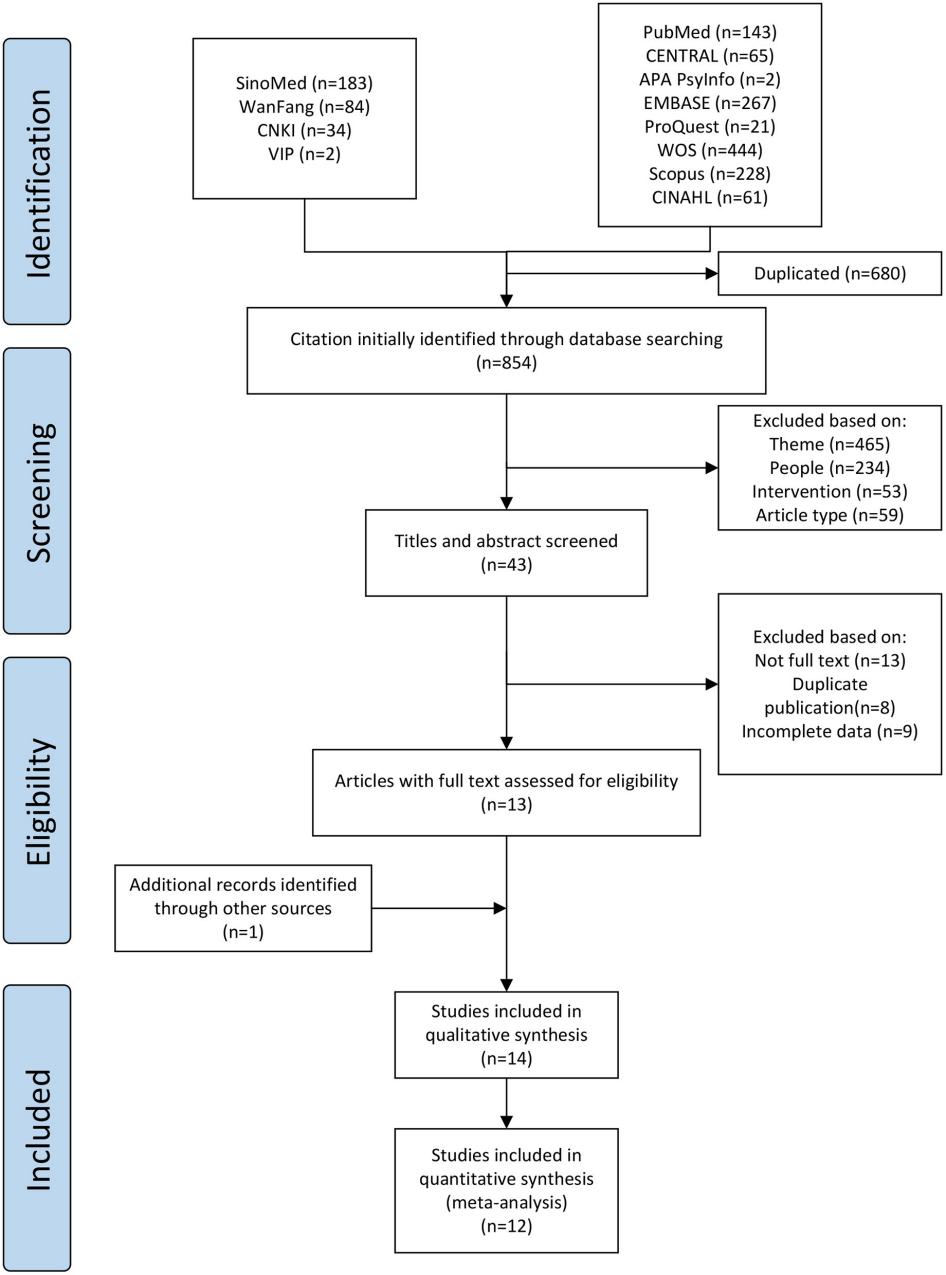
PRISMA flowchart of study selection.

Using the search strategy, 1,534 articles were identified from 12 databases (Figure 1). After eliminating duplicates through the reference management software and manual inspection, 854 articles remained. These 854 articles were then evaluated by screening their titles and abstracts; from them, 465 were not related to the research topic, 234 did not meet the participant criteria, 53 did not meet the intervention criteria, and 59 did not meet the criteria for the study type. Thus, based on the inclusion criteria, 43 studies were retained. After screening the full texts of these 43 articles, 13 were retained. One additional study was identified by evaluating the reference lists of the selected articles. These 14 studies were then included in the systematic review and meta-analysis.

### Characteristics of the Included Studies

The characteristics are presented in Table 1. A total of 980 participants were covered in these studies, of which 486 were allocated to the intervention groups and 494 to the control groups. These studies were published in English or Chinese and originated from various countries worldwide: China (*n* = 7) (Yi, 2015; Junmei, 2016; Xinjian, 2017; Zhu et al., 2018; Qi et al., 2019; Yu, 2019; Wang et al., 2020), USA (*n* = 2) (Barnes et al., 2013; Aguiñaga, 2016), Malaysia (*n* = 1) (Adam et al., 2016), Spain (*n* = 1) (Bisbe et al., 2020), Japan (*n* = 1) (Doi et al., 2017), Philippines (*n* = 1) (Dominguez et al., 2018), and Greece (*n* = 1) (Lazarou et al., 2017). Ten studies were RCTs (Barnes et al., 2013; Yi, 2015; Aguiñaga, 2016; Junmei, 2016; Doi et al., 2017; Lazarou et al., 2017; Zhu et al., 2018; Qi et al., 2019; Bisbe et al., 2020; Wang et al., 2020), and four studies (Adam et al., 2016; Xinjian, 2017; Dominguez et al., 2018; Yu, 2019) were quasi-experimental trials. Five studies compared a dance intervention to regular drug treatment or therapy (Barnes et al., 2013; Yi, 2015; Junmei, 2016; Xinjian, 2017; Dominguez et al., 2018); five compared dance interventions to educational programs (Doi et al., 2017; Zhu et al., 2018; Qi et al., 2019; Yu, 2019; Wang et al., 2020); two used randomized cross-over designs (Aguiñaga, 2016; Lazarou et al., 2017); one compared dance to physical therapy (Bisbe et al., 2020); and one compared dance to relaxation exercises (Adam et al., 2016). In the intervention group, the intervention time ranged from 6 to 48 weeks, and the intensity of dance was low to moderate, excluding four studies that did not provide data for the intensity (Adam et al., 2016; Doi et al., 2017; Lazarou et al., 2017; Dominguez et al., 2018). The intervention was performed 1 to 7 times each week, and the duration of the intervention ranged from 25 to 60 min.

Although the included studies were all based on aerobic dance forms, the dance style varied across studies; only two studies employed aerobic dance, three studies adopted ballroom dance as an intervention, three studies employed Chinese square dancing, two trials used unique dance forms (Dance Rug and Yangke), and other studies described their dance interventions as choreography, poco-poco dance, and standard dance-based aerobics format. The poco-poco dance course was guided by experienced professionals who gradually increased the difficulty and intensity of the dance both for acceptance and safety. BAILAMOS© program was professionally guided based on the BAILAMOS© guidebook. The researchers constantly adjusted the dance content in conjunction with the difficulty of the steps and safe limit of each of the participants. The dance used props (Velcro bracelets of different colors) to help participants remember their movements. Chinese square dancing, which originated in China, involves a variety of dance forms, but is also easy to learn. Dance rug is a modern aerobics game that is simple and easy to operate, allowing participants to learn at home without outside guidance. Yangko is a traditional Chinese folk-dance form. This dance mainly involves the twisting of various parts of the body; this unique form of exercise has particular advantages.

### Risk of Bias in the Included Studies

**Figure 2:**
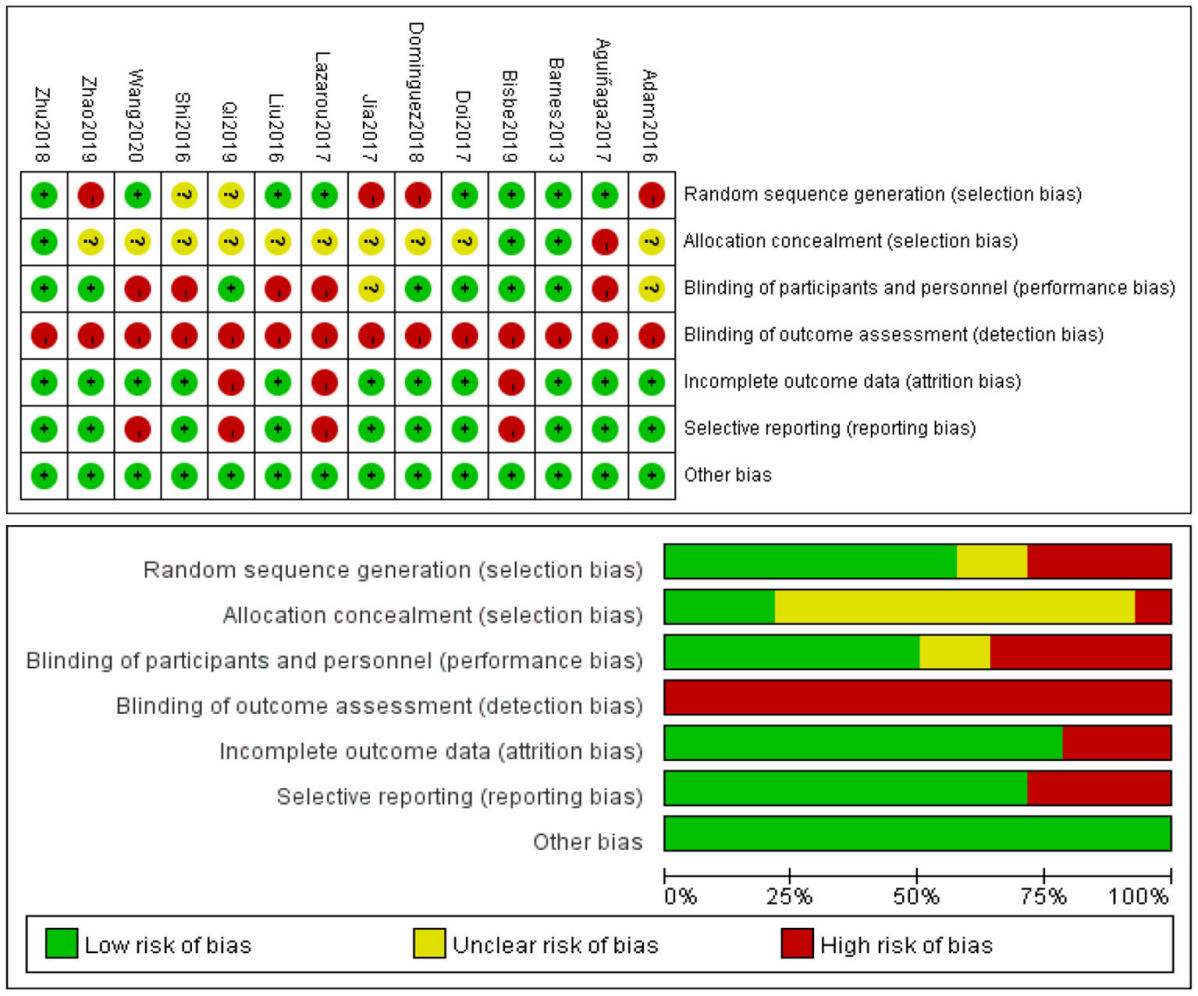
Results of Cochrane risk of bias tool.

The data for the risk of bias in the included studies is provided in Figure 2. Overall, the methodological quality of the trials was unsatisfactory, with all trials rated as having B quality. According to the Cochrane Handbook for Systematic Reviews of Interventions, all quasi-experimental trials were reported to show a high risk of bias. Only eight RCTs described the specific method of randomization, and only three of them reported allocation concealment. Five studies showed a high risk of bias since the participants and personnel were not blinded to the dance intervention. All studies demonstrated a high risk of detection bias because none of them provided information for the outcome assessment. Four studies had a high reporting bias, three of which did not provide a complete outcome statistic (Lazarou et al., 2017; Qi et al., 2019; Bisbe et al., 2020). Four studies had high reporting bias. The methodological quality of the trials was generally unsatisfactory (Lazarou et al., 2017; Qi et al., 2019; Bisbe et al., 2020; Wang et al., 2020).

### Effects of the Interventions

#### Primary Outcome: Cognitive Function

##### Global Cognition

**Figure 3:**
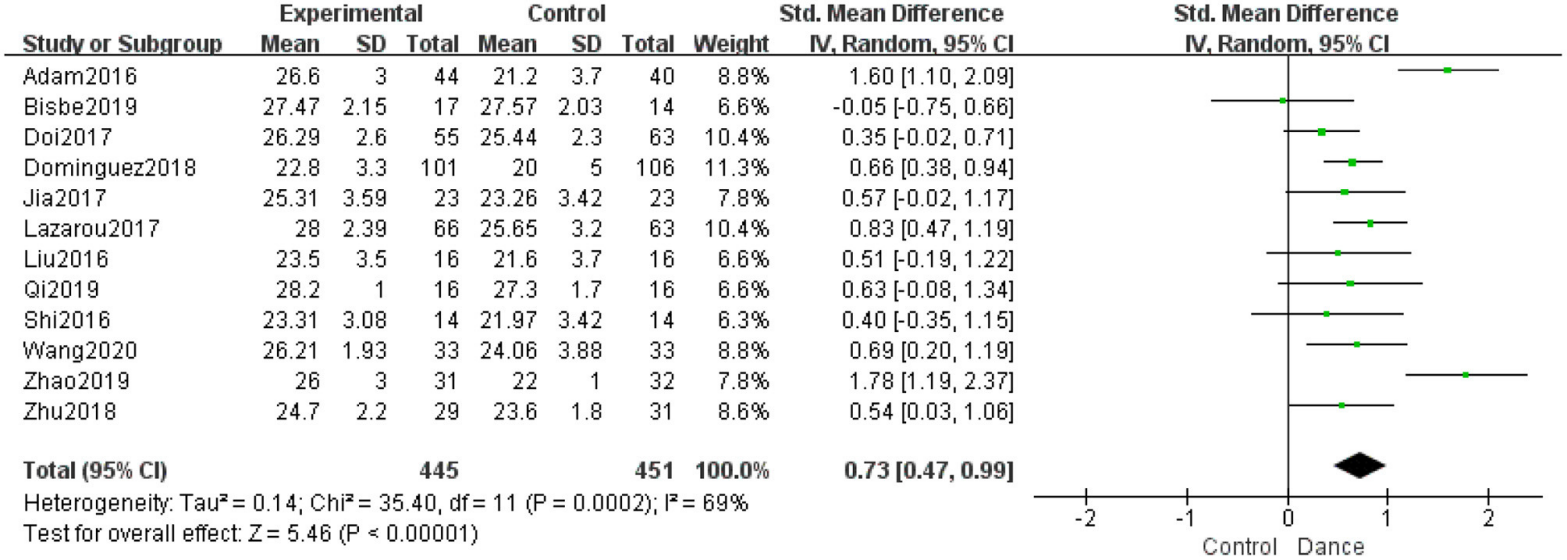
Forest plot of global cognition results of dance group vs. control group.

Twelve studies examined the effects of dance interventions on global cognition. Analysis of global cognition at the end of the intervention demonstrated a significantly higher post-intervention global cognition level in the dance group than in the control group. The pooled SMD showed a statistically significant increase in global cognition (SMD = 0.73, 95% CI: 0.47 to 0.99, *P* < 0.00001; Figure 3).

**Figure 4:**
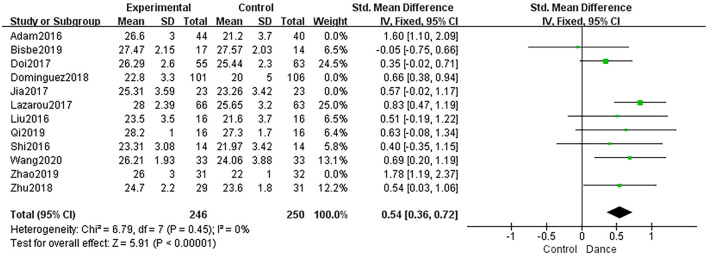
Forest plot of global cognition results of dance group vs. control group (sensitive analysis).

To analyze the source of the high heterogeneity (*I*^2^ = 69%, χ^2^ = 35.4, *P* = 0.0002), we performed a sensitivity analysis. When we excluded the four quasi-experimental trials (Adam et al., 2016; Xinjian, 2017; Dominguez et al., 2018; Yu, 2019), the *I*^2^ decreased from 69% to 0% without influencing the overall pooled effect (Figure 4).

##### Rote Memory

**Figure 5:**
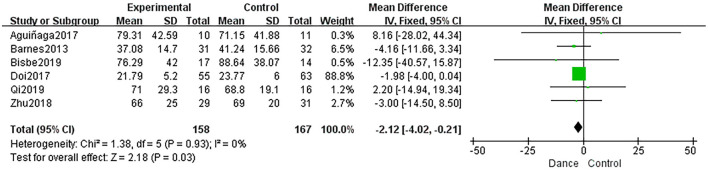
Forest plot of rote memory results of dance group vs. control group.

Six studies evaluated rote memory using the TMT-A. These studies were pooled for TMT-A results, and the results showed a statistically significant effect with no heterogeneity between studies (MD = −2.12, 95% CI: −4.02 to −0.21, *P* = 0.03, *I*^2^ = 0%; Figure 5).

##### Executive Function

**Figure 6:**
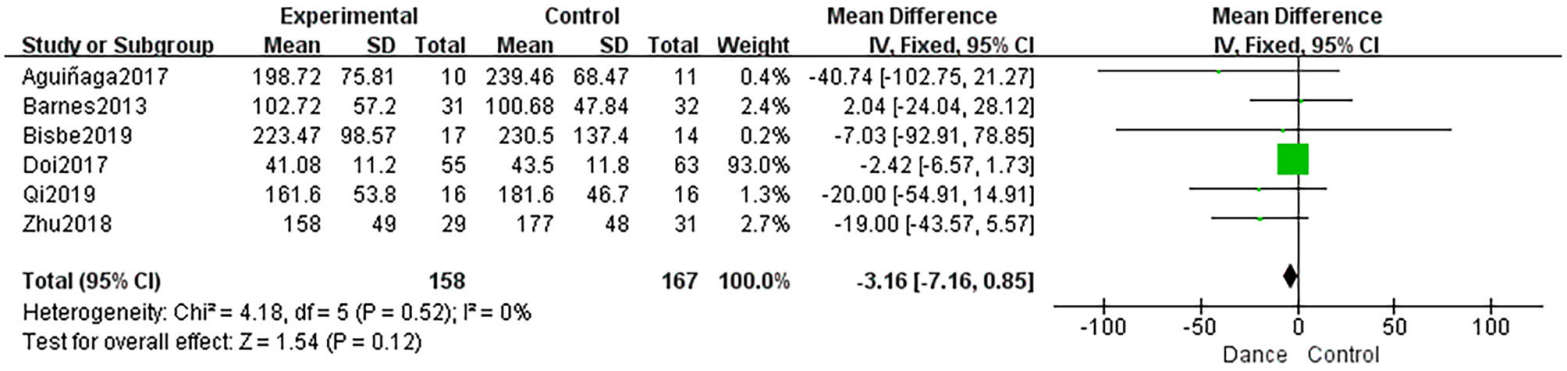
Forest plot of executive function results of dance group vs. control group.

The effect of dance interventions in improving executive function was examined in six studies (Barnes et al., 2013; Aguiñaga, 2016; Doi et al., 2017; Zhu et al., 2018; Qi et al., 2019; Bisbe et al., 2020). These studies were pooled for TMT-B scores, but they showed no significant changes (MD = −3.16, 95% CI: −7.16 to −0.85, *P* = 0.12; Figure 6).

##### Immediate Recall

**Figure 7:**
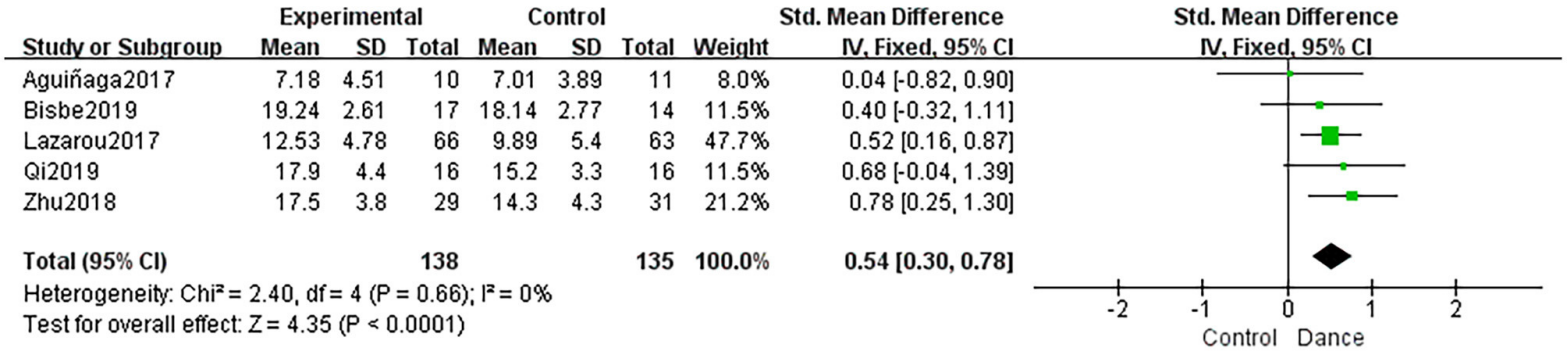
Forest plot of immediate recall results of dance group vs. control group.

Five studies used LM-1 and RBMT 1 to measure immediate recall, and the dance intervention group showed significantly improved immediate recall compared to the control group (SMD = 0.54, 95% CI: 0.30 to 0.78, *P* < 0.0001; *I*^2^ = 0%, χ^2^ = 2.40, *P* = 0.66; Figure 7).

##### Delayed Recall

**Figure 8:**
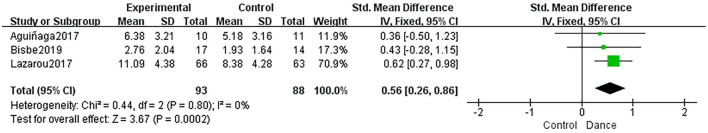
Forest plot of delayed recall results of dance group vs. control group.

Three studies investigated the effects of the dance intervention on delayed recall by obtaining LM-2 scores. The results indicated that dance intervention induced an improvement in delayed recall in comparison with the control group (SMD = 0.56, 95% CI: 0.26 to 0.86, *P* = 0.0002; Figure 8).

##### Attention

**Figure 9:**
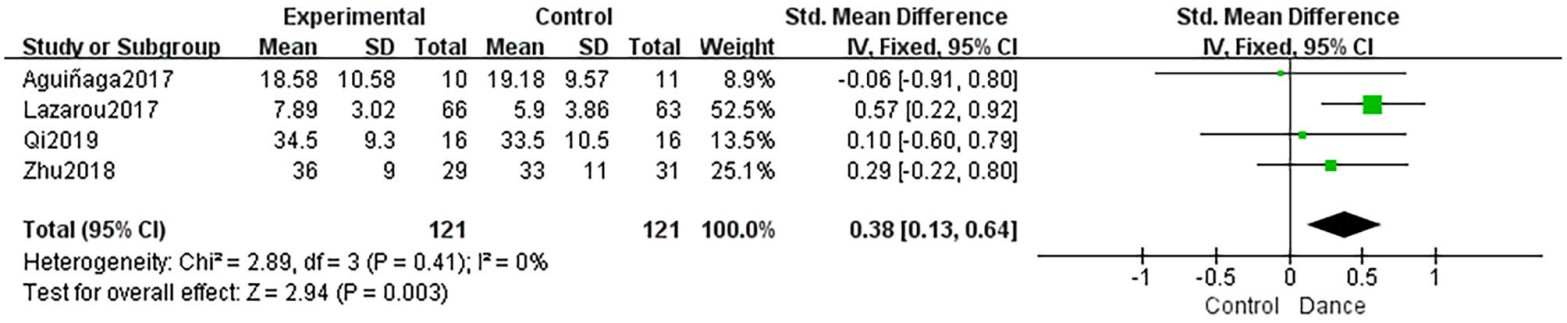
Forest plot of attention results of dance group vs. control group.

Four studies discussed the effect of dance interventions on attention using the SDMT or TEA. The dance intervention group showed significantly improved attention compared to the control group (SMD = 0.38, 95% CI: 0.13 to 0.64, *P* = 0.003; Figure 9).

##### Language

Two studies were pooled for the BNT results. One study illustrated that a structured modular ballroom dance intervention can improve language among older people with MCI (BNT score after treatment: dance group, 13.2 ± 1.3; control group, 12.6 ± 2.2; *P* < 0.05). Another study (Bisbe et al., 2020) found that greater language benefits were achieved with dance intervention than with physical therapy (BNT score after treatment: dance group, 45.71 ± 6.58; control group, 43.57 ± 5.96; *P* < 0.05).

#### Secondary Outcomes

##### Psycho-Behavioral Symptoms

###### Depression

**Figure 10:**
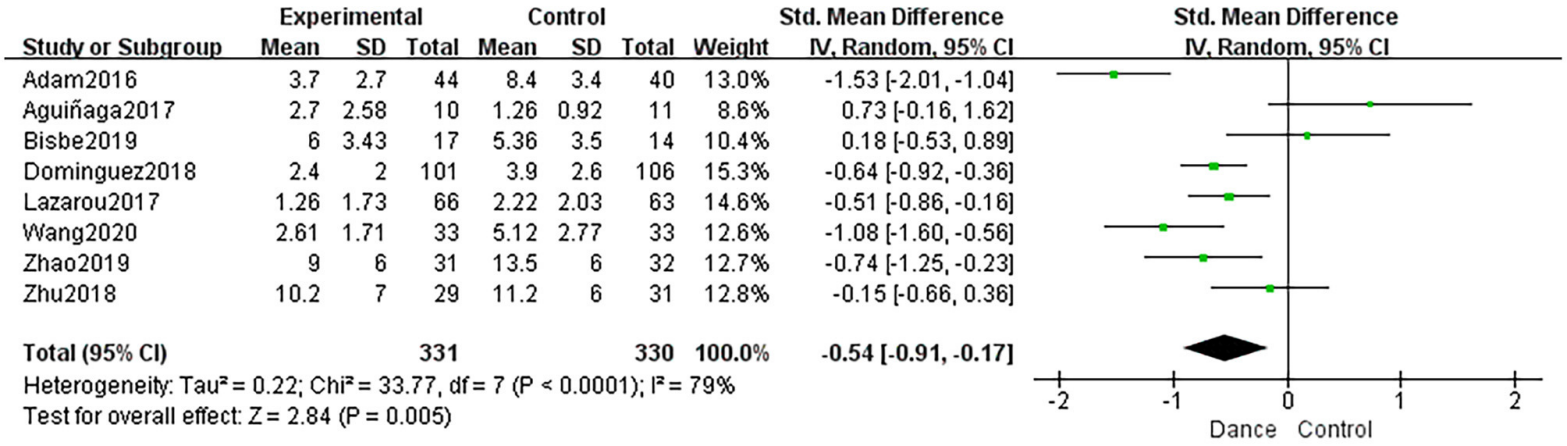
Forest plot of depression results of dance group vs. control group.

Eight studies assessed patients' depression. Because the pooled results exhibited high heterogeneity, subgroup analyses—according to the number of participants included and the scales used—were performed to search for sources of heterogeneity (*I* ^2^ = 79%, χ^2^ = 33.77, *P* < 0.0001; Figure 10). Therefore, our study only qualitatively described this outcome.

Eight studies compared the dance intervention group with non-dancing controls to evaluate the effectiveness of dance intervention on depression in 577 older adults. Two trials (Adam et al., 2016; Bisbe et al., 2020) used HADS to assess depression. One study (Adam et al., 2016) demonstrated that dance was more beneficial to treat depression than relaxation interventions alone (HADS score after treatment: dance group, 3.7 ± 2.7; control group, 8.4 ± 3.4; *P* < 0.01). However, another trial (Bisbe et al., 2020) reported no significant difference between the dance and control groups (HADS score after treatment: dance group, 6.0 ± 3.4; control group, 5.4 ± 3.5; *P* > 0.05). Four studies (Aguiñaga, 2016; Lazarou et al., 2017; Zhu et al., 2018; Wang et al., 2020) reported significant improvements in depression for the dance intervention (*P* < 0.05). One study (Yu, 2019) used GDS-30 and found an improvement in depression in the dance group (GDS-30 score after treatment: dance group, 10.2 ± 7.0; control group, 11.2 ± 6.0; *P* < 0.05). Another study (Dominguez et al., 2018), which only described the use of GDS to measure depression, found that dance intervention may help improve depression (GDS score after treatment: dance group, 2.4 ± 2.0; control group, 3.9 ± 2.6; *P* < 0.05).

##### Anxiety

Two studies (Adam et al., 2016; Bisbe et al., 2020) provided information on the effect of dance interventions on anxiety, as measured by the HADS. One study (Adam et al., 2016) demonstrated that dance was more beneficial for treatment of anxiety than relaxation interventions alone (HADS score after treatment: dance group, 4.4 ± 2.7; control group, 7.6 ± 3.1; *P* < 0.001). In contrast, another RCT (Aguiñaga, 2016) demonstrated that people in the dance group obtained higher HADS scores than those in the control group but without statistical difference (HADS score after treatment: dance group, 8.82 ± 3.25; control group, 5.43 ± 2.98; *P* > 0.05).

##### Dementia-Related Behavioral Symptoms

Two (Lazarou et al., 2017; Dominguez et al., 2018) of the 14 studies evaluated the effect of dance interventions on dementia-related behavioral symptoms as measured by the NPI. One trial found that international ballroom dancing had a positive effect in older adult patients with MCI (NPI score after treatment: dance group, 1.78 ± 2.28; control group, 3.76 ± 4.48; *P* > 0.05). Another study indicated that a 48-week ballroom dance program could improve these symptoms in older people with MCI (NPI score after treatment: dance group, 5 ± 9.3; control group, 5.4 ± 9.4; *P* > 0.05). However, both studies demonstrated no significant differences in dementia-rated behavioral symptoms between the two groups.

##### Motor Functions

###### Balance

**Figure 11:**
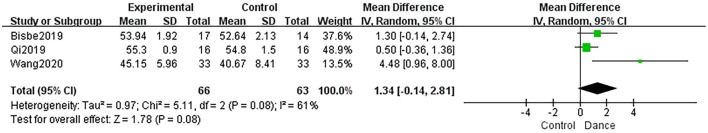
Forest plot of balance results of dance group vs. control group.

Three studies evaluated the effects on balance using the BBS, but found no significant differences (MD = 1.34, 95% CI: −0.14 to 2.81, *P* = 0.08; Figure 11). We did not perform a sensitivity analysis or subgroup analysis because of the limited number of studies.

##### Functional Mobility

Two studies (Aguiñaga, 2016; Bisbe et al., 2020) reported the findings for functional mobility, assessed by the TUG test. One RCT (Aguiñaga, 2016) demonstrated that people in the dance group obtained higher TUG scores than those in the control group (TUG score after treatment: dance group, 12.65 ± 3.01; control group, 11.99 ± 2.5; *P* < 0.05). Another RCT (Bisbe et al., 2020) demonstrated that dance was less beneficial for anxiety than treatment with physical therapy (TUG score after treatment: dance group, 8.15±1.37; control group, 9.01±1.75; *P* > 0.05).

##### Quality of Life

QOL scores were available for 136 participants from three trials. Two trials (Adam et al., 2016; Aguiñaga, 2016) reported QOL scores using QOL-AD assessments, and one trial (Bisbe et al., 2020) used the SF-36 physical functioning and mental functioning scores. One study that used QOL-AD (Adam et al., 2016) demonstrated that dance was more beneficial for QOL than treatment with a relaxation intervention alone (QOL-AD score after treatment: dance group, 36.4 ± 4.1; control group, 28.8 ± 4.4; *P* < 0.001). However, one RCT (Aguiñaga, 2016) used QOL-AD and showed that there was no significant difference between the dance and control groups (QOL-AD score after treatment: dance group, 38.82 ± 5.81; control group, 37.64 ± 5.7; *P* > 0.05). One study (Bisbe et al., 2020) uniquely measured QOL by determining the SF-36 score and reported no significant difference between the dance and control groups (SF-36 score after treatment: dance group, 99.76 ± 6.77; control group, 102.29 ± 4.41; *P* > 0.05).

## Discussion

### Summary of Findings

This systematic review evaluated the effects of dance interventions on cognitive functions, mental state, motor functions, and QOL in older adult patients with MCI. Fourteen studies were included, and the quality of the studies was graded as B. In general, the results of this meta-analysis revealed that dance interventions had a positive influence on older adult patients with MCI. The results of this data analysis strongly suggested that dance interventions, as a convenient intervention method, can effectively improve the global cognition, memory, attention and rote memory. Due to the different numbers of the literature included in each outcome index, the credibility of each outcome is different. For outcomes with fewer than three articles included, we performed descriptive analysis instead of meta-analysis (such as language, anxiety, behavioral symptoms related to dementia, functional mobility). Furthermore, we performed a descriptive analysis (such as depression, QOL) for those results of the meta-analysis with more significant heterogeneity (>50%).

The aggregate results of MMSE and MoCA evaluations showed that the dance intervention group was better than the control group in terms of overall cognition, and the results were durable and stable when sensitivity analysis was performed. Dance intervention as an aerobic exercise maintains the integrity of cerebral vessels by providing blood, oxygen, and nutrients and influences cognition by increasing brain-derived neurotrophic factor levels and synapses, promoting the growth and survival of neurons (Morais et al., 2018). The heterogeneity of the 12 included studies was significant. The sensitivity analysis showed that the type of study may be a source of heterogeneity, which reduced the credibility of these findings. Therefore, in addition to discussing the effects of overall cognitive improvement, we also discussed the effects of each cognitive field.

Immediate recall and delayed recall are used to quantify the memory function of patients with MCI and are the most common targets of cognitive impairment in MCI. Two published reviews have shown that dance interventions can promote immediate recall and delayed recall (Chan et al., 2020; Zhu et al., 2020), and our analysis showed similar results. Our study showed that the implementation of dance as an intervention often requires participants to first memorize the dance routine and then perform the corresponding dance movements with different types of music; this can be regarded as a type of memory training and may be the reason why dance interventions had a positive effect on memory function.

Dance interventions can also play a positive role in improving attention. The improvement in attention can be attributed to the consideration and integration of multiple senses (Ward et al., 2020). In dance activities, patients can improve attention through tactile stimulation provided by assistive devices in dancing, through auditory stimulation provided by music, and through visual stimulation provided by follow-up exercises. In the Oxford Dictionary, “dance” is defined as, “moving rhythmically to music, usually in a series of steps.” Rhythm perception is an essential ability to identify words and encode and decode language (Richter and Ostovar, 2016). Therefore, we considered that dance interventions can improve the language ability of patients by enhancing the sense of rhythm.

Zhu et al. (2020) used the verbal fluency test, TAT-A, and TMT-B to represent executive function, but the ability of the verbal fluency test to measure executive function remains uncertain (Whiteside et al., 2016), and there are subtle differences between TMT-A and TMT-B in their specific measurement purposes. Therefore, our study will discuss the measurement results of TMT-A and TMT-B separately.

TMT-A mainly measures rote memory (Llinàs-Reglà et al., 2017). Rote memory is a cognitive function that achieves memory effect through repeated learning related to external cues (Stevens and Bernier, 2013). Our comprehensive analysis showed that the improvement effect on rote memory in the dance group was better than that in the control group. This may be because music nodes and auxiliary bracelets were influential external cues in the dance intervention.

TMT-B mainly measures executive function (Llinàs-Reglà et al., 2017). Compared with TMT-A, it is more difficult for some patients to complete TMT-B. In the included studies, TMT-B was mainly used to evaluate the executive function of patients with MCI, which was quite tricky. Dance intervention cannot significantly improve the thinking flexibility of patients with MCI within a short time. In addition, executive function is closely related to the age and daily living ability of patients. The included participants of our study were elderly with MCI that had characteristics such as older age (≥60 years old), poor thinking flexibility, poor daily living ability, etc., and poor executive function is compared with the rest of the population, that was difficult to improve. Our aggregate results showed that dance intervention has no significant effect on executive function. In comparison with previous studies, the results of the meta-analysis of four RCT studies by Chan showed that dance intervention had no significant effect on the mental flexibility measured by TMT-B, which is consistent with the findings of our study. However, Zhu et al. (2020), who analyzed three RCTs, showed that dance interventions had a positive influence on the results of TMT-B assessments and the overall executive function, in contrast with our study. The main reason for these discrepancies may be the differences in inclusion criteria. Zhu's study defined Tai Chi as a dance form, which is debatable. Tai Chi focuses on the stability of exercise, not flexibility. At the same time, Tai Chi emphasizes the internalization of emotions, not the expression of emotions. Therefore, Tai Chi is not in line with our definition of dance intervention. In contrast, Zhu only included published RCTs but not quasi-experimental studies.

Depression, anxiety, and mental symptoms are fundamental reasons for the poor prognosis of older patients with MCI. Patients with MCI are reluctant to admit their illness for various reasons after diagnosis (Xanthopoulou and McCabe, 2019). The sense of shame and social loss of disease tends to increase depression and anxiety in patients with MCI. Simultaneously, the psychological state is closely related to cognitive function. An excellent psychological state can improve the cognitive function of patients (Li and Li, 2018).

Due to the high heterogeneity of the summary results, we conducted a qualitative analysis of the eight included studies. According to our result from the qualitative analysis, seven of eight studies were reported that dance intervention can improve depression in MCI patients better than the control intervention. But one study (Bisbe et al., 2020) showed the opposite result, and it might be related to the study's controlled intervention as physiotherapy. Based on these two conflicting results, we cannot yet assume that dance activity can improve the depression status of older patients with MCI. More original research is needed in the future to prove this result.

Although previous studies have shown that dance exercise can improve anxiety (Koch et al., 2019), the results of our study were not in concordance with this finding. Among the two included studies, one (Adam et al., 2016) was a quasi-experimental trial, and its results showed that in comparison with simple relaxation training, the combination of dance and relaxation was more helpful in improving the anxiety level of patients. Nevertheless, the anxiety level of patients was shown to not improve after a 12 week choreographed exercise intervention in another study (Bisbe et al., 2020). This may be because the control group in this study underwent physical therapy, which can also affect anxiety. Therefore, a summary of the existing studies could not clarify whether dance exercise effectively relieves anxiety, and more original studies are required to address this question.

After analyzing the results of two studies, we found that though the behavioral symptoms of the dance group improved compared to the control group, there was no significant difference in effect sizes across the dance group and the control group. Behavioral symptoms related to dementia include wandering, eating/toilet problems, delusions/hallucinations, aggressive/abusive speech, day and night reversal, excitement/tingling, apathy, depression/anxiety, violence, and high irritability (Tsunoda et al., 2020). The most common abnormal behavioral symptoms are depression/anxiety, which can significantly increase the incidence of dementia (Matsuoka et al., 2019). Although behavioral symptoms are not included in the diagnostic criteria of MCI, behavioral symptoms caused by anxiety, depression, and other emotions often impose great burdens on nurses and caregivers (Van der Mussele et al., 2013). It is unfortunate that the previous review did not discuss behavioral outcomes.

Falls are the leading cause of accidental injury and death in the older population (World Health Organization and Unit, 2008). Falls result from complex interactions between internal and external factors (Franco et al., 2020). Among internal factors, balance and mobility are core risk factors and preventive factors that can be altered (Giménez-Llort and Castillo-Mariqueo, 2020). Considering that the balance function of people with cognitive impairment is worse than that of healthy older adults, older people with MCI tend to have higher fall rates (Fuentes-Abolafio et al., 2021). Previous studies have found that dance involves visual control and the somatosensory and vestibular system to maintain balance (Filar-Mierzwa et al., 2020); thus, it can effectively improve the balance ability of older adults and reduce the incidence of falls (Shanahan et al., 2016; Filar-Mierzwa et al., 2017; Liu et al., 2021). However, a comprehensive analysis of the BBS score in our study showed that the balance ability of the dance group was not better than that in the control group. From the perspective of the measurement metric, the use of BBS as an indicator is more suited to evaluate the static balance ability, but existing literature finds that dance intervention affects the dynamic balance significantly more. Major dynamic balance measurements include the Tinetti Performance Oriented Mobility Assessment and TUG test (Tariq et al., 2006; Abreu and Hartley, 2013). Additionally, the National Institute for Clinical Evidence Research guidelines also advocate the use of the TUG test to assess gait and balance when preventing falls in older people (National Institute for Health Care Excellence, 2013). In the literature incorporated in our systematic review, the outcome of the TUG test measurements is described as functional mobility—a formulation of combining balance, gait, and mobility. Several studies have shown that although dance intervention improves the TUG score, it is not significant and is closely related to the duration of the intervention (Kaewjoho et al., 2020). The TUG test results in this study also showed that dance intervention was not significant for functional mobility improvement. However, from an intervention perspective, these findings may be related to significant differences between dance interventions. The effect of each dance style may vary by its rhythm and intensity, and the intervention time is usually short, which may also lead to inaccurate research results. Furthermore, differences in patient proficiency and dance experience may be another reason for changes in dance outcomes.

In a study of the nursing outcome preferences of patients with MCI, patients' QOL was shown to be the most significant result (Smith et al., 2018). QOL is a broad concept and includes subjective well-being and daily living conditions (Koch et al., 2019). QOL refers to one or some specific external characteristics and expresses the existing state of individuals. Therefore, its evaluation should focus on the subjective feelings of patients (Douka et al., 2019). Although the two scales are self-reported, they are primarily quantitative. At the same time, the implementation of dance intervention is progressive, and the improvement of participants' function is also from the shallow to the deep. Through the gradual advancement of their physical, mental, social, and spiritual aspects, the quality of life can be finally improved. The effects of dance intervention on the depression, anxiety and physical function of the elderly with MCI were unclear in the study we included. Moreover, there were significant differences in the typical values and explanations of different scales used to assess the QOL. These differences in measurements and the resultant heterogeneity in studies may make our evidence controversial.

### Strengths and Limitations

Our systematic review comprehensively explored the effects of dance intervention as a treatment on patients with MCI. All trials in this review have been conducted and published in the last 5 years, reflecting the importance of exploring the effects of dance intervention on patients with MCI in recent years. Considering the rapid development of dance intervention studies in China, our study has incorporated more of Chinese literature compared to previous studies. Although there are currently systematic reviews and meta-analyses of the effects of dance intervention on cognitive function in patients with MCI, our systematic review and meta-analysis may be the first to explore the effects of dance intervention on motor function, psycho-behavioral symptoms, and QOL of older people with MCI.

However, there are several limitations to this review. First, since dance intervention is a new non-drug intervention, there have been few intervention studies so far, and the studies we have included were medium quality, affecting the credibility of our review. A substantial number of literature reports did not include certain results, so we could not perform a meta-analysis on them and the final results need to be treated with caution. Furthermore, due to the heterogeneity of the measurement tools for the type of dance intervention, the setting of the intervention, and the partial outcome indicator, we were unable to perform a meta-analysis in its entirety. Finally, the review includes only the literature written in both Chinese and English; thus, some studies reported in other languages may have been missed.

### Implications for Practice and Research

Overall, this review supports previous findings that older people with MCI can benefit from various dance interventions. At this point, the evidence strongly supports the management of dance in global cognition, rote memory, immediate memory, delayed memory and attention. More studies are needed to determine the effects of dance on other cognitive functions, psycho-behavioral symptoms, motor functions and QOL. The quality of the studies that we included was medium, which indicates that there is still room for methodological progress in existing dance intervention studies. Future studies should specify recruitment methods and recruit participants more systematically to enhance sample representation, take appropriate controls to improve the internal effectiveness of the findings, clearly describe the number and reasons for withdrawals and exits, and adopt creative approaches to improve compliance. In addition, we found that existing studies had fewer long-term follow-up studies and that future studies could extend follow-up time to observe the long-term effects of dance interventions in the population and the population's dependence on dance interventions.

There is no uniform standard for dance intervention in the current study, nor is there any recommended frequency, intensity, time, type, and other intervention characteristics. Future studies can be conducted from two aspects: first, we can compare the characteristics of different levels of intervention and conduct RCTs in the population to arrive at better recommendations for intervention levels. Second, because there are a variety of dances, patient-centered dance intervention programs can be formulated according to the patient's actual situation, personal preferences, and physical and mental conditions.

Finally, in terms of outcome indicators, future studies should first standardize the measurement methods of different endings and ensure the measurement methods' authentic measurement results and significance. At the same time, because dance experience has a significant subjective component, future studies can include more qualitative methods to establish a more comprehensive assessment of the effects of dance.

## Conclusion

In conclusion, our meta-analysis showed that dance interventions may positively affect cognitive function, rote memory, immediate recall, delayed recall and attention in patients with MCI. However, the included study does not indicate that dance intervention had positive results on the improvement in executive function and balance in patients with MCI. Moreover, with the fewer studies included or high study heterogeneity, the effect of dance intervention on language, dementia-related behavioral symptoms, depression, anxiety, functional mobility, and QOL in patients with MCI needs to be further verified. Current studies have evaluated various types of dance interventions. However, the foundation of the intervention methods is weak, and the possibility of randomization bias is high. Nevertheless, there are a few high-quality studies on this topic. These encouraging results need to be carried forward with strictly controlled study designs to continue to verify and expand the comprehensive application effect of dance intervention on patients with MCI.

## Data Availability Statement

The original contributions presented in the study are included in the article/Supplementary Material, further inquiries can be directed to the corresponding author/s.

## Author Contributions

CL and MS conducted the literature search and extracted and interpreted data. CL wrote the first draft of the manuscript. CL, MS, and YJia revised the manuscript. YJi and SZ provided suggestions on writing and revised the article. All authors read and approved the final manuscript.

## Funding

This work was supported by Nursing project of superior discipline construction in Jiangsu Universities (szbf [2018] No. 87), Nursing science, a key discipline project of Jiangsu Province during the 13th Five-Year Plan period (sjy [2016] No. 9), Project Studies on Construction of Core Competency Model and Development of Assessment Tool for Nurses of Hospice Care supported by NSFC (72004099), and Project Comparative study on hospice care mode between China and Canada (2017SJB0295) supported by Philosophy and Social Science Foundation of universities in Jiangsu Province.

## Conflict of Interest

The authors declare that the research was conducted in the absence of any commercial or financial relationships that could be construed as a potential conflict of interest.

## Publisher's Note

All claims expressed in this article are solely those of the authors and do not necessarily represent those of their affiliated organizations, or those of the publisher, the editors and the reviewers. Any product that may be evaluated in this article, or claim that may be made by its manufacturer, is not guaranteed or endorsed by the publisher.
