# Supplementary material for: Effects of Dance Interventions on Cognition, Psycho-Behavioral Symptoms, Motor Functions, and Quality of Life in Older Adult Patients With Mild Cognitive Impairment: A Meta-Analysis and Systematic Review
Source: Front Aging Neurosci. 2021 Sep 20;13:706609. doi: 10.3389/fnagi.2021.706609 (PMC8488360; doi:10.3389/fnagi.2021.706609)
Supplement: Supplementary file 1 [file Data_Sheet_1.DOCX]

Supplementary Material

# Search Strategy

**PubMed**

| Search | Query | Results |
| --- | --- | --- |
| #1 | "cognitive dysfunction"[MeSH Terms] | 19135 |
| #2 | ((((("mild cognitive impairment*"[Title/Abstract]) OR ("cognitive dysfunction"[Title/Abstract])) OR ("cognitive declin*"[Title/Abstract])) OR ("cognitive impairment*"[Title/Abstract])) OR ("mild neurocognitive disorder*"[Title/Abstract])) OR ("mental deterioration*"[Title/Abstract]) | 95193 |
| #3 | #1 OR #2 | 98956 |
| #4 | "dancing"[MeSH Terms] | 2933 |
| #5 | "dance therapy"[MeSH Terms] | 378 |
| #6 | "danc*"[Title/Abstract] OR "Salsa"[Title/Abstract] OR "Rumba"[Title/Abstract] OR "Waltz"[Title/Abstract] OR "Cha Cha"[Title/Abstract] OR "Blues"[Title/Abstract] OR "Jitterbug"[Title/Abstract] OR "Tango"[Title/Abstract] OR "Viennese"[Title/Abstract] OR "Foxtrot"[Title/Abstract] OR "Swing"[Title/Abstract] OR "Merengue"[Title/Abstract] OR "ballet"[Title/Abstract] OR "Disco"[Title/Abstract] | 20318 |
| #7 | #4 OR #5 OR #6 | 20839 |
| #8 | #3 AND #7 | 143 |

**Cochrane Library**

| Search | Query | Results |
| --- | --- | --- |
| #1 | MeSH descriptor:[Cognitive Dysfunction] explode all trees | 1491 |
| #2 | (“mild cognitive impairment*”):ti, ab, kw | 2842 |
| #3 | (“cognitive dysfunction*”):ti, ab, kw | 3182 |
| #4 | (“cognitive impairment*”):ti, ab, kw | 7986 |
| #5 | (“cognitive decline”):ti, ab, kw | 2738 |
| #6 | (“mild neurocognitive disorder*”):ti, ab, kw | 42 |
| #7 | (“mental deterioration*”):ti, ab, kw | 198 |
| #8 | #1 OR #2 OR #3 OR #4 OR #5 OR #6 OR #7 | 11864 |
| #9 | MeSH descriptor:[Dancing] explode all trees | 171 |
| #10 | MeSH descriptor:[Dance Therapy] explode all trees | 85 |
| #11 | (danc*):ti, ab, kw | 1086 |
| #12 | (Salsa):ti, ab, kw | 33 |
| #13 | (Rumba):ti, ab, kw | 9 |
| #14 | (Waltz):ti, ab, kw | 16 |
| #15 | (cha-cha):ti, ab, kw | 6 |
| #16 | (Blues):ti, ab, kw | 152 |
| #17 | (Jitterbug):ti, ab, kw | 0 |
| #18 | (Tango):ti, ab, kw | 117 |
| #19 | (Viennese):ti, ab, kw | 21 |
| #20 | (Foxtrot):ti, ab, kw | 25 |
| #21 | (Swing):ti, ab, kw | 749 |
| #22 | (Merengue):ti, ab, kw | 0 |
| #23 | (ballet):ti, ab, kw | 62 |
| #24 | #9 OR #10 OR #11 OR #12 OR #13 OR #14 OR #15 OR #16 OR #17 OR #18 OR #19 OR #20 OR #21 OR #22 OR #23 | 2151 |
| #25 | #8 AND #24 | 65 |

**APA PsyInfo**

| Search | Query | Results |
| --- | --- | --- |
| #1 | MeSH: (cognitive dysfunction) | 70 |
| #2 | Title: "mild cognitive impairment*" OR Abstract: "mild cognitive impairment*" OR Title: "cognitive dysfunction*" OR Abstract: "cognitive dysfunction*" OR Title: "cognitive impairment*" OR Abstract: "cognitive impairment*" OR Title: "cognitive declin*" OR Abstract: "cognitive declin*" OR Title: "mental deterioration*" OR Abstract: "mental deterioration*" OR Title: "mild neurocognitive disorder*" OR Abstract: "mild neurocognitive disorder*" | 876 |
| #3 | #1 OR #2 | 898 |
| #4 | MeSH: Dancing | 11 |
| #5 | MeSH: dance therapy | 0 |
| #6 | Title: danc* OR Abstract: danc* OR Title: salsa OR Abstract: salsa OR Title: rumba OR Abstract: rumba OR Title: waltz OR Abstract: waltz OR Title: cha-cha OR Abstract: cha-cha OR Title: blues OR Abstract: blues OR Title: jitterbug OR Abstract: jitterbug OR Title: tango OR Abstract: tango OR Title: viennese OR Abstract: viennese OR Title: foxtrot OR Abstract: foxtrot OR Title: swing OR Abstract: swing OR Title: merengue OR Abstract: merengue OR Title: ballet OR Abstract: ballet | 297 |
| #7 | #4 OR #5 OR #6 | 298 |
| #8 | #3 AND #7 | 2 |

**EMBASE**

| Search | Query | Results |
| --- | --- | --- |
| #1 | 'mild cognitive impairment'/exp | 27815 |
| #2 | 'mild cognitive impairment*':ti,ab | 27976 |
| #3 | 'cognitive dysfunction*':ti,ab | 23123 |
| #4 | 'cognitive impairment*':ti,ab | 103414 |
| #5 | 'cognitive declin*':ti,ab | 36340 |
| #6 | 'mild neurocognitive disorder*':ti,ab | 304 |
| #7 | 'mental deterioration*':ti,ab | 1466 |
| #8 | #1 OR #2 OR #3 OR #4 OR #5 OR #6 OR #7 | 150507 |
| #9 | 'dancing'/exp | 5166 |
| #10 | 'dance therapy'/exp | 513 |
| #11 | 'danc*':ti,ab | 9750 |
| #12 | 'salsa':ti,ab | 839 |
| #13 | 'rumba':ti,ab | 31 |
| #14 | 'waltz':ti,ab | 231 |
| #15 | 'cha-cha':ti,ab | 49 |
| #16 | 'blues':ti,ab | 2128 |
| #17 | 'jitterbug':ti,ab | 16 |
| #18 | 'tango':ti,ab | 1169 |
| #19 | 'viennese':ti,ab | 657 |
| #20 | 'foxtrot':ti,ab | 66 |
| #21 | 'swing':ti,ab | 10288 |
| #22 | 'merengue':ti,ab | 6 |
| #23 | 'ballet':ti,ab | 1355 |
| #24 | #9 OR #10 OR #11 OR #12 OR #13 OR #14 OR #15 OR #16 OR #17 OR #18 OR #19 OR #20 OR #21 OR #22 OR #23 | 26249 |
| #25 | #8 AND #24 | 267 |

**ProQuest**

| Search | Query | Results |
| --- | --- | --- |
| #1 | ti("mild cognitive impairment*") OR ab("mild cognitive impairment*") OR ti(("cognitive dysfunction")) OR ab(("cognitive dysfunction")) OR ti(("cognitive impairment" OR "cognitive impairments")) OR ab(("cognitive impairment" OR "cognitive impairments")) OR ti(“cognitive decline”) OR ab(“cognitive decline”) OR ti(“mental deterioration*”) OR ab(“mental deterioration*”) OR ti(“mild neurocognitive disorder*”) OR ab(“mild neurocognitive disorder*”) | 5742 |
| #2 | ti(“danc*”) OR ab(“danc*”) OR ti(“salsa”) OR ab(“salsa”) OR ti(“rumba”) OR ab(“rumba”) OR ti(“waltz”) OR ab(“waltz”) OR ti(“cha-cha”) OR ab(“cha-cha”) OR ti(“blues”) OR ab(“blues”) OR ti(“jitterbug”) OR ab(“jitterbug”) OR ti(“tango”) OR ab(“tango”) OR ti(“viennese”) OR ab(“viennese”) OR ti(“foxtrot”) OR ab(“foxtrot”) OR ti(“swing”) OR ab(“swing”) OR ti(“merengue”) OR ab(“merengue”) OR ti(“ballet”) OR ab(“ballet”) | 18040 |
| #3 | #1 AND #2 | 21 |

**WOS**

| Search | Query | Results |
| --- | --- | --- |
| #1 | TS=("mild cognitive impairment*") OR TS=("cognitive dysfunction*") OR TS=("cognitive impairment*") OR TS=("cognitive declin*") OR TS=("mild neurocognitive disorder*") OR TS=("mental deterioration*") | 126943 |
| #2 | TS=(danc*) OR TS=(salsa) OR TS=(rumba) OR TS=(waltz) OR TS=(cha-cha) OR TS=(blues) OR TS=(jitterbug) OR TS=(tango) OR TS=(viennese) OR TS=(foxtrot) OR TS=(swing) OR TS=(merengue) OR TS=(ballet) | 391020 |
| #3 | #1 AND #2 | 444 |

**Scopus**

| Search | Query | Results |
| --- | --- | --- |
| #1 | (TITLE-ABS-KEY("mild cognitive impairment*")  OR TITLE-ABS-KEY ("cognitive dysfunction*") OR TITLE-ABS-KEY ("cognitive impairment*") OR TITLE-ABS-KEY ("cognitive declin*") OR TITLE-ABS-KEY ("mild neurocognitive disorder*") OR TITLE-ABS-KEY ("mental deterioration*")) | 122363 |
| #2 | ( TITLE-ABS-KEY (danc*) OR TITLE-ABS-KEY (salsa) OR TITLE-ABS-KEY (rumba) OR TITLE-ABS-KEY (waltz) OR TITLE-ABS-KEY (cha-cha) OR TITLE-ABS-KEY (blues) OR TITLE-ABS-KEY (jitterbug) OR TITLE-ABS-KEY (tango) OR TITLE-ABS-KEY (viennese) OR TITLE-ABS-KEY (foxtrot) OR TITLE-ABS-KEY (swing) OR TITLE-ABS-KEY (merengue) OR TITLE-ABS-KEY (ballet)) | 107092 |
| #3 | #1 AND #2 | 228 |

**CINAHL**

| Search | Query | Results |
| --- | --- | --- |
| #1 | (MM "Mild Cognitive Impairment") | 82 |
| #2 | TI "mild cognitive impairment*" OR AB "mild cognitive impairment*" OR TI "cognitive dysfunction*" OR AB "cognitive dysfunction*" OR TI "cognitive impairment*" OR AB "cognitive impairment*" OR TI “cognitive declin*” OR AB “cognitive declin*” OR TI “mental deterioration*” OR AB “mental deterioration*” OR TI “mild neurocognitive disorder*” OR AB “mild neurocognitive disorder*” | 21926 |
| #3 | #1 OR #2 | 32897 |
| #4 | (MH "Dancing+") OR (MH "Dance Therapy") | 4590 |
| #5 | TI danc* OR AB danc* OR TI salsa OR AB salsa OR TI rumba OR AB rumba OR TI waltz OR AB waltz OR TI cha-cha OR TI cha-cha OR TI blues OR AB blues OR TI jitterbug OR AB jitterbug OR TI tango OR AB tango OR TI viennese OR AB viennese OR TI foxtrot OR AB foxtrot OR TI swing OR AB swing OR TI merengue OR AB merengue OR TI ballet OR AB ballet | 11012 |
| #6 | #4 OR #5 | 11543 |
| #7 | #3 AND #6 | 61 |

**SinoMed**

| Search | Query | Results |
| --- | --- | --- |
| #1 | "认知障碍"[加权:扩展] | 12725 |
| #2 | "轻度认知障碍"[中文标题:智能] OR "轻度认知障碍"[摘要:智能] OR "认知下降"[中文标题:智能] OR "认知下降"[摘要:智能] OR "认知障碍"[中文标题:智能] OR "认知障碍"[摘要:智能] | 23418 |
| #3 | (#1) OR (#2) | 25218 |
| #4 | "舞蹈疗法"[不加权:扩展] | 14 |
| #5 | "舞蹈"[不加权:扩展] | 551 |
| #6 | "舞"[中文标题:智能] OR "舞"[摘要:智能] OR "芭蕾"[中文标题:智能] OR "芭蕾"[摘要:智能] OR "拉丁"[中文标题:智能] OR "拉丁"[摘要:智能] OR "国标"[中文标题:智能] OR "国标"[摘要:智能] | 10661 |
| #7 | (#4) OR (#5) OR (#6) | 10666 |
| #8 | (#3) AND (#7) | 183 |

**Wangfang**

| Search | Query | Results |
| --- | --- | --- |
| #1 | 主题:("认知障碍") | 22232 |
| #2 | 题名或关键词:("轻度认知障碍") or 题名或关键词:(认知障碍) or 题名或关键词:(认知下降) | 47365 |
| #3 | #1 OR #2  主题:("认知障碍") OR 题名或关键词:("轻度认知障碍") or 题名或关键词:(认知障碍) or 题名或关键词:(认知下降) | 50955 |
| #4 | 主题:("舞蹈") or 主题:("舞蹈疗法") | 107006 |
| #5 | 题名或关键词:("舞") or 题名或关键词:("芭蕾") or 题名或关键词:("拉丁") or 题名或关键词:("国标") | 318151 |
| #6 | #4 OR #5  题名或关键词:("舞") or 题名或关键词:("芭蕾") or 题名或关键词:("拉丁") or 题名或关键词:("国标") OR 主题:("舞蹈") or 主题:("舞蹈疗法") | 331853 |
| #7 | #3 AND #6 | 84 |

**CNKI**

| Search | Query | Results |
| --- | --- | --- |
| #1 | (主题=认知障碍) | 14385 |
| #2 | (篇关摘=轻度认知障碍) OR (篇关摘=认知下降) OR (篇关摘=认知障碍) | 3087 |
| #3 | #1 OR #2  (主题=认知障碍) OR (篇关摘=轻度认知障碍) OR (篇关摘=认知下降) OR (篇关摘=认知障碍) | 159695 |
| #4 | (主题=舞蹈) OR (主题=舞蹈疗法) | 100071 |
| #5 | (篇关摘=舞) OR (篇关摘=芭蕾) OR (篇关摘=拉丁) OR (篇关摘=国标) | 183767 |
| #6 | #4 OR #5  (主题=舞蹈) OR (主题=舞蹈疗法) OR (篇关摘=舞) OR (篇关摘=芭蕾) OR (篇关摘=拉丁) OR (篇关摘=国标) | 275165 |
| #7 | #3 AND #6  ((主题=认知障碍) OR (篇关摘=轻度认知障碍) OR (篇关摘=认知下降) OR (篇关摘=认知障碍)) AND ((主题=舞蹈) OR (主题=舞蹈疗法) OR (篇关摘=舞) OR (篇关摘=芭蕾) OR (篇关摘=拉丁) OR (篇关摘=国标)) | 34 |

**VIP**

| Search | Query | Results |
| --- | --- | --- |
| #1 | ((题名或关键词=轻度认知障碍 OR (((((题名或关键词=认知障碍 OR 题名或关键词=cognition disorders) OR 题名或关键词=cognitive deficit) OR 题名或关键词=cognitive disorder) OR 题名或关键词=cognitive disorders) OR 题名或关键词=神经行为障碍)) OR 题名或关键词=认知下降) | 4451 |
| #2 | (((题名或关键词=舞 OR 题名或关键词=芭蕾) OR 题名或关键词=拉丁) OR 题名或关键词=国标) | 1081 |
| #3 | #1 OR #2 | 2 |

# Quality assessment of methodology of included studies

| **TABLE 2 \|** Quality assessment of methodology of included studies. | | | | | | | | |
| --- | --- | --- | --- | --- | --- | --- | --- | --- |
| References | Sequence generation | Allocation concealment | Blinding | | Incomplete outcome data | Selective outcome reporting | Other sources of bias | Level |
|  |  |  | Therapist and participants | Outcome assessors |  |  |  |  |
| Adam2016 | High risk | Unclear | Unclear | High risk | Low risk | Low risk | Low risk | B |
| Dominguez2018 | High risk | Unclear | Low risk | High risk | Low risk | Low risk | Low risk | B |
| Aguiñaga2017 | Low risk | High risk | High risk | High risk | Low risk | Low risk | Low risk | B |
| Barnes2013 | Low risk | Low risk | Low risk | High risk | Low risk | Low risk | Low risk | B |
| Bisbe2019 | Low risk | Low risk | Low risk | High risk | High risk | High risk | Low risk | B |
| Doi2017 | Low risk | Unclear | Low risk | High risk | Low risk | Low risk | Low risk | B |
| Lazarou2017 | Low risk | Unclear | High risk | High risk | High risk | High risk | Low risk | B |
| Qi2019 | Unclear | Unclear | Low risk | High risk | High risk | High risk | Low risk | B |
| Wang2020 | Low risk | Unclear | High risk | High risk | Low risk | High risk | Low risk | B |
| Zhu2018 | Low risk | Low risk | Low risk | High risk | Low risk | Low risk | Low risk | B |
| Liu2016 | Low risk | Unclear | High risk | High risk | Low risk | Low risk | Low risk | B |
| Shi2016 | Unclear | Unclear | High risk | High risk | Low risk | Low risk | Low risk | B |
| Jia2017 | High risk | Unclear | Unclear | High risk | Low risk | Low risk | Low risk | B |
| Zhao2019 | High risk | Unclear | Low risk | High risk | Low risk | Low risk | Low risk | B |
